# Acute gout attacks during the perioperative period and risk factors of recurrence after orthopedic surgery among untreated gout patients

**DOI:** 10.1186/s13018-023-03536-8

**Published:** 2023-01-23

**Authors:** Hui Wang, Chao Yan, Qiping Wu, Hao Zeng, Zhihong Zhang, Wanming Wang, Xiaotang Sun

**Affiliations:** 1 Department of Orthopedics Surgery, The 900th Hospital of Joint Logistic Support Force, PLA, 156 West Second Ring North Road, Gulou District, Fuzhou, 350025 Fujian Province People’s Republic of China; 2 Fuzong Clinical Medical College of Fujian Medical University, Fuzhou, 350025 Fujian Province People’s Republic of China; 3 School of Health Care, MinJiang Teachers College, Fuzhou, 350108 Fujian Province People’s Republic of China

**Keywords:** Uricemia, Gout, Acute attack, Orthopedics, Perioperative period

## Abstract

**Background:**

This study aimed to explore the clinical characteristics of perioperative acute gout attacks in patients with varying uric acid levels undergoing orthopedic surgery, identify the risk factors for gout recurrence within the first postoperative year, and provide a disease prevention and diagnostic reference.

**Methods:**

This hospital-based retrospective study was conducted between January 2018 and December 2020. According to the blood uric acid levels at admission, the patients were grouped into either the normal uric acid level group or the hyperuricemia group. Patient comorbidities, serum uric acid levels, inflammatory indicators, follow-up recurrence rates, and other indicators were compared.

**Result:**

The uric acid decline ratio and the inflammatory indexes (white blood cell count and C-reactive protein level) at the time of the attack were significantly higher in the normal uric acid level group than in the hyperuricemia group (*P* < 0.05). Patients in the hyperuricemia group with diabetes and tophi and those administered diuretics were more prone to acute gout attacks than those in the normal uric acid level group (*P* < 0.05). In the normal uric acid level group, 22 patients (84.6%) exhibited single joint involvement, whereas only 18 patients (47.4%) in the hyperuricemia group demonstrated single joint involvement (*P* < 0.05). After 1 year of follow-up, the gout recurrence rate in the hyperuricemia group was 44.7%, which was significantly higher that the recurrence rate in the normoglycemic group (11.5%; *P* < 0.05). Presenting tophi in perioperative orthopedic surgery patients was found to be an independent risk factor for gout recurrence within 1 year (RR = 4.80; *P* = 0.029).

**Conclusion:**

The recurrence rate of gout in patients with hyperuricemia during perioperative period increased 1 year after operation. Therefore, it is crucial to monitor the uric acid level to prevent acute gout attacks during the perioperative period and recurrence during the 1-year follow-up period. Moreover, the risk of an acute gout recurrence 1 year after operation increased in patients who presented tophi; therefore, it is necessary to maintain appropriate blood uric acid level during perioperative period among patients undergoing orthopedic surgery.

**Supplementary Information:**

The online version contains supplementary material available at 10.1186/s13018-023-03536-8.

## Introduction

Gout is a recurrent type of inflammatory arthritis caused by the deposition of urate crystals in joints or soft tissues [1]. The prevalence of gout has been increasing, causing a heavy medical and economic burden and seriously affecting the health-related quality of life [2]. Gout attacks are very painful and are typically characterized by the sudden onset of severe and intense joint pain. Factors that may precipitate a gout attack include alcohol intake, a high-purine diet, trauma, and surgery [3, 4]. A study at Boston University reported that in 633 patients with gout, acute purine intake led to a fivefold increase in the risk of a recurrent gout attack. The impact from animal purine sources was substantially greater than that from plant purine sources [4]. A study of 724 patients in the USA reported that patients with gout had a fourfold increased risk of gout attacks during hospitalization [5].

Notably, surgery is believed to predispose patients to gout attacks. Moreover, perioperative gout may interfere with patient diagnoses, possibly resulting in the misdiagnosis of an infectious disease [6], delaying the patient's operation and/or postoperative recovery, and increasing the patient's medical treatment costs [5, 7]. Therefore, in several respects, early detection and management of perioperative gout are important.

The diagnosis of an acute gout attack is based primarily on typical clinical manifestations, the presence of hyperuricemia, and/or the presence of monosodium urate (MSU) crystals in synovial fluid analysis. However, 11–49% of patients maintain normal serum uric acid (UA) levels during acute gout attacks [8–10], which suggests that, in addition to UA, there are hidden risk factors affecting the recurrence of gout. Therefore, in this study, we investigated the effects of different UA levels on acute perioperative gout attacks and recurrences within the first postoperative year in patients undergoing orthopedic surgery.

## Methods

### Population

This retrospective analysis of acute perioperative gout attacks involved patients hospitalized in the Orthopedics Department of our hospital between January 2018 and December 2020. The inclusion criteria required each patient to meet the 2015 American College of Rheumatology/European League Against Rheumatism classification and diagnostic criteria for gout [11]. At least one episode of joint swelling and pain was necessary for initial diagnosis. Consequently, the presence of tophi or MSU crystals in the joint or synovial fluid was a sufficient condition for gout diagnosis. If this sufficient condition was not met, clinical signs, laboratory test findings, and imaging were used. A cumulative score of laboratory and imaging studies with ≥ 8 points was used to clinically diagnose patients with gout. In addition, patients were only included if they had complete hospitalization and follow-up data, were aged between 18 and 75 years at the time of the gout attack, and were undergoing orthopedic surgery. Patients were excluded if they had an active bacterial infection (including tuberculosis); pseudogout; comorbidities of severe heart, lung, liver, and/or kidney dysfunction; evidence of rheumatoid or connective tissue disease; or administration of allopurinol within 6 months.

In this study, no UA-lowering therapy was required in the acute phase. The treatment plan for patients with gout after discharge was non-steroidal anti-inflammatory drugs (NSAIDs) administration combined with sodium bicarbonate to alkalinize the urine. If the drug did not relieve symptoms after 5 days, glucocorticoid treatment (metoprolol 4 mg) was administered.

All investigations were approved by the ethic committee of the 900th Hospital of the Joint Logistics Team, PLA (No. 2022-017 on July 8, 2022), and informed consent was obtained from all participants.

### Data collection

The age, sex, body mass index (BMI), and UA level data for each patient were recorded at admission, during the acute attack, and upon remission. Additionally, we also extracted information from each patient’s medical record regarding the time and duration of the acute gout attack; febrile response; the number of involved joints; inflammatory indexes (white blood cell count [WBC], c-reactive protein [CRP] level, and erythrocyte sedimentation rate [ESR]) at the time of the acute attack and upon resolution; smoking and drinking histories; history of medications (diuretics and aspirin); presence/absence of tophi; and presence/absence of hypertension, hyperlipidemia, diabetes, coronary heart disease, and kidney disease. Follow-up was also performed approximately 1 year after discharge to determine whether the patient had additional acute gout attacks within that 1 year. Once a patient developed gout-like symptoms during the follow-up process, the follow-up staff arranged for the patient to arrive at the hospital for further examination, thus ensuring the reliability of the follow-up results.

### Definitions

In this study, an acute gout attack was defined as the onset of symptoms and signs of gout within 3 weeks of hospitalization. Based on the 2015 American College of Rheumatology/European League Against Rheumatism classification criteria for gout [1], musculoskeletal ultrasonography at the attack site was used to confirm the presence of at least one of the following positive signs in the synovium or cartilage: (1) urate crystals, tophi, and double tracks; (2) significant thickening of the synovial membrane on the affected side compared with the contralateral side; or (3) a depression defect on the surface of the cartilage.

Hyperuricemia was defined as a serum UA level of ≥ 416 µmol/L (7.0 mg/dL) in men or ≥ 357 µmol/L (6.0 mg/dL) in women [1]. According to the serum UA level at admission, the patients were divided into either the normal UA level group or hyperuricemia group. The UA decline ratio was defined as follows: (the serum UA level at admission minus the level at the time of the acute attack)/serum UA level at admission**.** The time from admission to gout attack was defined as gout induction time (day).

### Statistical analysis

Continuous variables (age, BMI, UA, WBC, CRP, and ESR) are presented as means and standard deviations, and between-group comparisons were performed using Student’s *t* test. Categorical variables are presented as numbers and frequencies, with between-group comparisons being calculated using Chi-squared tests. Logistic regression analyses were used to explore the predictors of gout attacks, and the results are expressed as adjusted relative risks (RRs) and 95% confidence intervals (CIs). In this study, we used the Shapiro–Wilk (S–W) test to test the normality of the data. All analyses were conducted using SPSS for Windows (Version 25.0; SPSS, Chicago, IL, USA). *P* value < 0.05 was considered statistically significant.

## Results

**Fig. 1:**
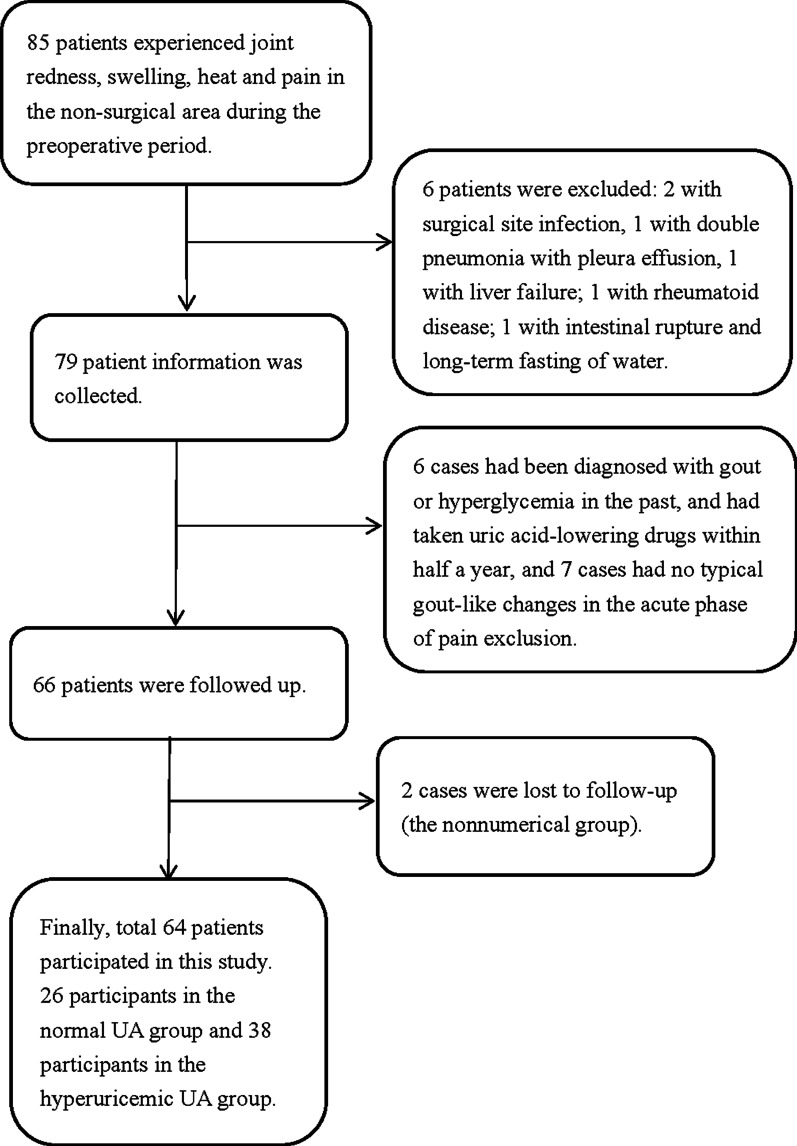
Participants’ recruitment flowchart

A total of 64 patients experienced acute gout attacks during the perioperative period and were included in this study, including 26 in the normal UA level group and 38 in the hyperuricemia group (Fig. 1). In this study, 6 patients had acute gout attacks before the operation (2 patients in the normal uricemia group and 4 patients in the hyperuricemia group), and 58 patients developed acute gout attacks after the operation (24 patients in the normal uricemia group and 34 patients in the hyperuricemia group). The patients with an acute attack before the operation were patients with fractures. The patients with gout attack after the operation were patients who underwent orthopedic surgeries such as fracture reduction and internal fixation, joint replacement, and spinal decompression.

The admission UA level in the hyperuricemia and normal uricemia groups was 546.45 ± 78.91 μmol/L and 319.67 ± 62.23 μmol/L, respectively. There were no significant differences in the mean age of the patients (*P* = 0.553), sex ratio (*P* = 0.801), or BMI (*P* = 0.649) between the groups (Table 1). At the onset of the acute gout attack, the majority of the patients in the normal UA level group demonstrated single joint involvement (*n* = 22; 84.6%), whereas the majority of those in the hyperuricemia group demonstrated multi-joint involvement (*n* = 20; 52.6%), which was significantly different between the two groups (*P* = 0.003). In addition, the two groups demonstrated different proportions of patients demonstrating fever, with the proportion of patients with fever in the hyperuricemia group (*n* = 12; 46.2%) being higher than that in the normal UA level group (*n* = 10, 26.3%); however, there was no significant difference between the two groups (*P* = 0.101). The duration of local pain, redness, and swelling caused by the acute gout attack was generally shorter in the normal UA level group (4.46 ± 1.94 days) than in the hyperuricemia group (5.55 ± 2.74 days), but the difference was not statistically significant (Table 1).

**Table 1:** The difference between the normal UA group and the hyperuricemic group in untreated acute gout patients

Category	Normal UA group (*n* = 26)	Hyperuricemic group (*n* = 38)	*P*
Age, means ± SD, year	54.96 ± 2.20	52.90 ± 16.54	0.553
Male, *n* (%)	22 (84.6)	33(86.8)	0.801
BMI, means ± SD, Kg/m^2^	24.11 ± 3.12	24.50 ± 3.54	0.649
Gout induction time, means ± SD, day	2.50 ± 1.39	2.74 ± 0.37	0.641
Admission uric acid, means ± SD, μmol/L	319.67 ± 62.23	546.45 ± 78.91	< 0.001
Gout attack uric acid, means ± SD, μmol/L	231.08 ± 97.91	442.96 ± 110.45	< 0.001
Gout relieves uric acid, means ± SD, μmol/L	277.28 ± 48.01	418.96 ± 80.08	< 0.001
Uric acid drop ratio (%)	42.12 ± 19.52	22.65 ± 10.24	< 0.001
Gout duration, means ± SD, day	4.46 ± 1.94	5.55 ± 2.74	0.085
Inflammatory markers during gout attack, means ± SD			
WBC (10^9^/L)	12.17 ± 4.42	10.23 ± 2.18	0.047
CRP (mg/dl)	138.36 ± 86.58	85.83 ± 47.15	0.008
ESR (mm/h)	74.65 ± 38.12	65.67 ± 32.20	0.313
Inflammatory markers in gout remission, means ± SD			
WBC (10^9^/L)	9.27 ± 2.67	8.07 ± 2.53	0.075
CRP (mg/dl)	54.73 ± 53.26	45.16 ± 37.27	0.400
ESR (mm/h)	57.67 ± 21.25	49.97 ± 23.09	0.181
Fever, *n* (%)	12 (46.2)	10 (26.3)	0.101
Hypertension, *n* (%)	12 (46.2)	18 (47.4)	0.924
Hyperlipidemia, *n* (%)	16 (61.5)	23 (60.5)	0.935
Diabetes, *n* (%)	3 (11.5)	15 (39.5)	0.015
Coronary heart disease, *n* (%)	10 (38.5)	12 (31.6)	0.569
Renal insufficiency, *n* (%)	2 (7.7)	6 (15.8)	0.336
Smoking, *n* (%)	8 (30.8)	15 (39.5)	0.476
Tophi, *n* (%)	2 (7.7)	20 (52.6)	< 0.001
Diuretics, *n* (%)	4 (15.4)	19 (50.0)	0.005
Drinking, *n* (%)	10 (38.5)	9 (23.7)	0.204
Aspirin, *n* (%)	5 (19.2)	9 (23.7)	0.672
Affected joints, n (%)			0.003
Single joint	22 (84.6)	18 (47.4)	
Multi-joint	4 (15.4)	20 (52.6)	

*SD* standard deviation, *BMI* body mass index, *WBC* white blood cell, *CRP* C-reactive protein, *ESR* erythrocyte sedimentation rate

The uric acid drop ratio in the normal UA level group (42.12 ± 19.52) was significantly higher than that in the hyperuricemia group (22.65 ± 10.24, *P* < 0.001). Additionally, the normal UA level group (WBC: 12.17 ± 4.42 10^9^/L; CRP: 138.36 ± 86.58 mg/dl) had significantly higher inflammatory index values (WBC and CRP levels) during the acute gout attack than the hyperuricemia group (WBC: 10.23 ± 2.18 10^9^/L; CRP: 85.83 ± 47.15 mg/dL) (WBC: *P* = 0.047 and CRP: *P* = 0.008, respectively). When the gout symptoms resolved, however, the inflammatory indexes were similar in both groups (all *P* > 0.05). Moreover, the prevalence of diabetes, diuretic use, and tophi was significantly higher in the hyperuricemia group than in the normal UA level group (all *P* > 0.05) (Table 1).

The average follow-up time for the patients in the normal UA level group and hyperuricemia group was 17.81 ± 10.36 and 21.92 ± 13.33 months, respectively. The gout recurrence rate was significantly higher (*n* = 17; 44.7%) in the hyperuricemia group than in the normal UA level group (*n* = 3; 11.5%, *P* = 0.005; Table 2). The patients in this study were grouped according to the serum UA level at admission. When the grouping criteria was a serum UA level of 300 µmol/L, there was no significant difference in the recurrence rate of gout after 1 year. However, the 1-year recurrence rate of gout in the group with serum UA levels ≤ 420 µmol/L and ≤ 360 µmol/L at admission was significantly higher than that in the group with UA levels > 420 µmol/L and > 360 µmol/L (Additional file 1: Table S1). Therefore, it may be safer for a patient to have a preoperative serum UA level below 300 µmol/L.

**Table 2:** Comparison of gout recurrence rate between normal uric acid group and hyperuricemic group within 1 year

Category	Normal UA group (*n* = 26)	Hyperuricemic group (*n* = 38)	*P*
Follow-up time, means ± SD, month	17.81 ± 10.36	21.92 ± 13.33	0.171
Recurrence of gout within 1 year, *n* (%)	3 (11.5)	17 (44.7)	0.005

Univariate analysis showed that there were significant differences between the gout recurrence group and the no gout recurrence group in UA level grouping (*P* = 0.005), hyperlipidemia (*P* = 0.035), renal insufficiency (*P* = 0.041), presence of tophi (*P* < 0.001), BMI (*P* = 0.031), and uric acid drop ratio (*P* = 0.011) (Table 3).

**Table 3:** Univariate analysis of the influencing factors of gout recurrence

Category	Gout recurrence		*P*
	Yes	No	
Age, means ± SD, year	56.2 ± 15.54	52.61 ± 14.11	0.365
Gender, *n* (%)			0.252
Male	19 (34.5)	36 (65.5)	
Female	1 (11.1)	8 (88.9)	
BMI, means ± SD, Kg/m^2^	25.68 ± 3.88	23.73 ± 2.94	0.031
BMI group, *n* (%)			0.090
No obesity (BMI < 28 kg/m^2^)	15 (27.3)	40 (72.7)	
Obesity (BMI ≥ 28 kg/m^2^)	5 (55.6)	4 (44.4)	
Gout attack uric acid, means ± SD, μmol/L	409.26 ± 137.95	333.08 ± 146.48	0.054
Gout relieves uric acid, means ± SD, μmol/L	394.79 ± 100.86	346.22 ± 93.89	0.066
Uric acid drop ratio, means ± SD	0.22 ± 0.11	0.34 ± 0.19	0.011
Inflammatory markers during gout attack, means ± SD:			
WBC (10^9^/L)	11.56 ± 2.61	10.77 ± 3.69	0.396
CRP (mg/dl)	118.37 ± 58.04	117.99 ± 77.76	0.985
ESR (mm/h)	74.45 ± 41.33	66.99 ± 31.52	0.430
Inflammatory markers in gout remission, means ± SD			
WBC (10^9^/L)	8.75 ± 2.64	8.47 ± 2.66	0.701
CRP (mg/dl)	52.42 ± 34.08	47.52 ± 48.54	0.685
ESR (mm/h)	55.6 ± 19.84	51.97 ± 23.76	0.554
Fever, *n* (%)	8 (40.0)	14 (31.8)	0.523
Hypertension, *n* (%)	9 (45.0)	21 (47.7)	0.839
Hyperuricemic, *n* (%)	17 (85.0)	21 (47.7)	0.005
Hyperlipidemia, *n* (%)	16 (80.0)	23 (52.3)	0.035
Diabetes, *n* (%)	7 (35.0)	11 (25.0)	0.410
Coronary heart disease, *n* (%)	7 (35.0)	15 (34.1)	0.943
Renal insufficiency, *n* (%)	5 (25.0)	3 (6.8)	0.041
Smoking, *n* (%)	10 (50.0)	13 (29.5)	0.114
Tophi, *n* (%)	14 (70.0)	8 (18.2)	< 0.001
Diuretics, *n* (%)	10 (50.0)	13 (29.5)	0.114
Drinking, *n* (%)	5 (25.0)	14 (31.8)	0.580
Aspirin, *n* (%)	5 (25.0)	9 (20.5)	0.683
Affected joints, *n* (%)			0.884
Single joint	14 (70.0)	30 (68.2)	
Multi-joint	6 (30.0)	14 (31.8)	

*SD* standard deviation, *BMI* body mass index, *WBC* white blood cell, *CRP* C-reactive protein, *ESR* erythrocyte sedimentation rate

After adjusting for UA level grouping, hyperlipidemia, renal insufficiency, presence of tophi, BMI, and UA decline ratio, tophi were associated with gout recurrence within the first year after their acute perioperative attack, with RR of 4.80 (95% CI, 1.18–19.55; *P* = 0.029) (Table 4). Although the risk of gout recurrence was higher in the hyperuricemia group than in the normal UA level group, the difference was not statistically significant (*P* = 0.406; Table 4). Therefore, presenting tophi was the independent predictor of gout recurrence within the first year after orthopedic surgery.

**Table 4:** Analysis of independent risk factors for gout recurrence

Category	Reference	RR	95% CI	*P*
Hyperuricemia group	No	2.07	0.37–11.48	0.406
Hyperlipidemia	No	2.01	0.21–19.05	0.544
Tophi	No	4.80	1.18–19.55	0.029
Renal insufficiency	No	2.96	0.36–24.45	0.315
BMI	–	1.05	0.77–1.44	0.739
Uric acid drop ratio	–	0.05	0.00–10.18	0.272

## Discussion

This follow-up study explores the different clinical characteristics of patients undergoing orthopedic surgery who suffered from acute perioperative gout attacks and identifies the risk factors for gout recurrence. In the normal uric acid level group, the UA level fluctuation was greater than in the hyperuricemia group and the inflammatory reaction was stronger than in the hyperuricemia group when the acute gout attack occurred during the perioperative period. The patients in the hyperuricemia group had a higher mean acute gout recurrence rate during the follow-up period. Presenting tophi was the independent predictor of gout recurrence within the first year after orthopedic surgery. Moreover, it may be safe for a patient to have a preoperative serum UA level below 300 µmol/L.

During acute gout attacks, the serum UA levels for patients in both groups decreased to varying degrees compared with the UA level at the time of admission; the serum UA levels decreased by 42.12% in the normal uric acid group and by 22.65% in the hyperuricemia group. However, there was more fluctuation in the UA levels in the normal UA level group than in the hyperuricemia group. This study showed that blood levels of inflammatory markers (WBC and CRP levels) were higher in the normal UA group patients than in hyperuricemia group patients during the perioperative acute gout attack. Li et al. followed the UA levels in patients for 8 years and found that patients with more obvious fluctuations in UA levels demonstrated higher risks of all-cause mortality and cardiovascular disease, potentially related to the high inflammatory response induced by UA level fluctuations [12]. However, the relationship between inflammation and UA levels is clinically controversial. Viveros-Paredes et al. [13] reported that inflammatory factors released into the blood circulation from tissues during acute gout attacks are positively correlated with changes in blood cortisol levels. Severe inflammatory responses stimulate cortisol secretion, thereby stimulating UA excretion. A decrease in serum UA levels can also activate the release of a series of inflammatory cytokines. Furthermore, UA level fluctuations can cause partial dissolution of preexisting tophi, activating a series of pro-inflammatory cytokines, which ultimately leads to acute gout attacks [14]. Hyperuricemia may induce the expression of hepatic inflammatory molecules by activating the pro-inflammatory NF-κB signaling cascade [15]. However, after multivariate analysis, serum cortisol levels in patients with essential hypertension were negatively correlated with eGFRcr-cys; however, serum cortisol levels in patients with essential hypertension were not negatively correlated with uric acid [16].

During the follow-up of the patients in this study, the gout recurrence rate in the hyperuricemia group was 44.7% within the first postoperative year, which was significantly higher than that in the normal UA level group. Spiga et al. [17] showed that persistent hyperuricemia leads to acute gout attacks by stimulating the production of inflammatory factors, such as interleukin-6 and tumor necrosis factor-α, or by inducing a systemic inflammatory response through the nuclear factor-kappa B signaling pathway. This also partly explains the high recurrence rate of gout in the hyperuricemia group 1 year after surgery in this study.

Obesity is the most important risk factor for developing gout [18]. The potential causal relationship between obesity, serum UA levels, and gout risk is plausible [19], and weight loss interventions can effectively reduce gout attacks. At the population level, a large number of prospective cohort studies related to BMI have shown that lowering a patient’s BMI has an important effect on reducing the risk of gout [20, 21]. In the present study, BMI was associated with gout recurrence in the univariate analysis. However, BMI was not an independent factor influencing gout recurrence after adjusted covariables.

Tophi are the result of long-term uncontrolled uric acid. Serum urate (sUA) concentrations above the solubility limit can lead to crystal deposits [22]. Over time, patients with gout who maintain an sUA below 6 mg/dL (360 μmol/L) can expect to remain gout-free [23, 24], but higher sUA levels can cause gout stone formation and an increased risk of gout recurrence [25, 26]. The formation of gout stones is evidence of poor control of uric acid levels over a long period of time, and elevated uric acid levels are a major risk factor for gout attacks [27]; therefore, gout stone formation indirectly causes an increased risk of recurrent gout in patients. Consistent with previous studies, the present results of this study showed that Tophi was an independent risk factor for gout recurrence within 1 year.

In our study, all patients experienced some degree of joint swelling and pain, and 34.4% (22/68) of patients had a fever. After treatment measures, including the administration of NSAIDs, drinking > 2000 mL/day of water, and glucocorticoid administration (metoprolol 4 mg), the symptoms were relieved after 4–5 days. However, when the gout attack site is the surgical site, especially in a joint that has been replaced [28, 29], the most important differential diagnosis is periprosthetic infection. Both diseases can manifest as joint redness, swelling, heat, pain, dysfunction, and the presence of elevated inflammatory marker levels. The main indicators of gout are (1) medical history, focusing on previous gout attacks; (2) detailed physical examinations to determine whether there is pain in other parts of the body, such as joints that are prone to gout (first metatarsophalangeal joint and metacarpophalangeal joint); (3) accurate tests (e.g., local musculoskeletal ultrasound, dual-energy computed tomography, joint cavity punctures, joint fluid leukocyte counts, classification, bacterial cultures, and urate crystal observations); and (4) the results of attempts to diagnose and treat (if the symptoms are significantly relieved following the administration of NSAIDs, colchicine, or other drugs, a diagnosis of gout is likely).

Antibiotics are not recommended as a treatment course until a definitive diagnosis is made, as they can affect the overall management of periprosthetic infections. An involved joint may demonstrate both gout and infection. Although rare and mostly reported in individual cases [30], a previous study [31] reported 30 cases of infected joints, wherein the local deposition of urate crystals was observed while culturing the pathogenic bacteria. Therefore, detailed medical history inquiries and careful physical and related examinations can reduce misdiagnoses.

This study has some limitations. First, the serum UA measurements during the perioperative period were not made under standard conditions. In clinical workups, blood can generally be drawn from patients on an empty stomach, without special concerns about whether the patient has had a high-purine diet. The diet has a certain degree of influence, resulting in index deviation. Second, normal patients under stress conditions, such as trauma and surgery, exhibit inflammatory markers (WBC, CRP, ESR, and others) that peak within 24–72 h after the stress incident. At this time, the measured inflammatory indicators may not accurately reflect the acute gout attack. The results of this study need to be verified in future studies with larger sample sizes.

## Conclusion

During acute gout attacks in the perioperative period of orthopedic surgery, the UA level fluctuations in patients in the normal uric acid level group are more obvious and the inflammatory response is stronger than in patients in the hyperuricemia group. The hyperuricemia group has a greater proportion of patients with gout than the normal UA level group. It is safer for a patient to maintain a preoperative serum UA level below 300 µmol/L. The recurrence rate of gout in patients with hyperuricemia during perioperative period increased 1 year after operation. It is crucial to monitor the uric acid level to prevent acute gout attacks during the perioperative period and recurrence during the 1-year follow-up period. Moreover, the risk of gout recurrence increased in patients who presented tophi; therefore, it is necessary to maintain appropriate blood uric acid level during perioperative period among patients undergoing orthopedic surgery.

## Supplementary Information

**Additional file 1**. **Supplement Table 1.** Effects of different uric acid groups on gout attack.

## Data Availability

The datasets generated during and/or analyzed during the current study are available from the corresponding author on reasonable request.

## Abbreviations

UA: Uric acid
BMI: Body mass index
WBC: White blood cell count
CRP: C-reactive protein
ESR: Erythrocyte sedimentation rate
SDs: Standard deviations
RRs: Relative risks
Cis: Confidence intervals

## References

[1] Dalbeth N, Gosling AL, Gaffo A, Abhishek A (2021). Gout. Lancet.

[2] Arromdee E, Michet CJ, Crowson CS, O'Fallon WM, Gabriel SE (2002). Epidemiology of gout: is the incidence rising. J Rheumatol.

[3] Kang EH, Lee EY, Lee YJ, Song YW, Lee EB (2008). Clinical features and risk factors of postsurgical gout. Ann Rheum Dis.

[4] Zhang Y, Chen C, Choi H (2012). Purine-rich foods intake and recurrent gout attacks. Ann Rheum Dis.

[5] Dubreuil M, Neogi T, Chen CA (2013). Increased risk of recurrent gout attacks with hospitalization. Am J Med.

[6] Oldmeadow LB, Edwards ER, Kimmel LA, Kipen E, Robertson VJ, Bailey MJ (2006). No rest for the wounded: early ambulation after hip surgery accelerates recovery. ANZ J Surg.

[7] Rothenbacher D, Primatesta P, Ferreira A, Cea-Soriano L, Rodríguez LA (2011). Frequency and risk factors of gout flares in a large population-based cohort of incident gout. Rheumatology (Oxford).

[8] Zhang WZ (2021). Why does hyperuricemia not necessarily induce gout. Biomolecules.

[9] Bardin T, Richette P (2014). Definition of hyperuricemia and gouty conditions. Curr Opin Rheumatol.

[10] Lin KC, Lin HY, Chou P (2000). The interaction between uric acid level and other risk factors on the development of gout among asymptomatic hyperuricemic men in a prospective study. J Rheumatol.

[11] Neogi T, Jansen TL, Dalbeth N (2015). 2015 gout classification criteria: an American College of Rheumatology/European League Against Rheumatism collaborative initiative. Ann Rheum Dis.

[12] Li S, Cui L, Cheng J (2020). Repeated measurements of serum urate and mortality: a prospective cohort study of 152,358 individuals over 8 years of follow-up. Arthritis Res Ther.

[13] Viveros-Paredes JM, Puebla-Pérez AM, Gutiérrez-Coronado O, Sandoval-Ramírez L, Villaseñor-García MM (2006). Dysregulation of the Th1/Th2 cytokine profile is associated with immunosuppression induced by hypothalamic-pituitary-adrenal axis activation in mice. Int Immunopharmacol.

[14] Liang S, Zhang D, Qi J, Song X, Xue J (2018). Reduced peak stimulated growth hormone is associated with hyperuricemia in obese children and adolescents. Sci Rep.

[15] Spiga R, Marini MA, Mancuso E (2017). Uric acid is associated with inflammatory biomarkers and induces inflammation via activating the NF-κB signaling pathway in HepG2 cells. Arterioscler Thromb Vasc Biol.

[16] Li X, Xiang X, Hu J (2016). Association between serum cortisol and chronic kidney disease in patients with essential hypertension. Kidney Blood Press Res.

[17] Chhana A, Pool B, Wei Y (2019). Human cartilage homogenates influence the crystallization of monosodium urate and inflammatory response to monosodium urate crystals: a potential link between osteoarthritis and gout. Arthritis Rheumatol.

[18] McCormick N, Rai SK, Lu N, Yokose C, Curhan GC, Choi HK (2020). Estimation of primary prevention of gout in men through modification of obesity and other key lifestyle factors. JAMA Netw Open.

[19] Robinson PC, Stamp LK (2016). The management of gout: much has changed. Aust Fam Phys.

[20] Nielsen SM, Bartels EM, Henriksen M (2017). Weight loss for overweight and obese individuals with gout: a systematic review of longitudinal studies. Ann Rheum Dis.

[21] Maglio C, Peltonen M, Neovius M (2017). Effects of bariatric surgery on gout incidence in the Swedish Obese Subjects study: a non-randomised, prospective, controlled intervention trial. Ann Rheum Dis.

[22] Tausche AK, Manger B, Müller-Ladner U, Schmidt B (2012). Gout as a systemic disease. Manifestations, complications and comorbidities of hyperuricaemia. Z Rheumatol.

[23] Zhang W, Doherty M, Bardin T (2006). EULAR evidence based recommendations for gout. Part II: management. Report of a task force of the EULAR Standing Committee for International Clinical Studies Including Therapeutics (ESCISIT). Ann Rheum Dis.

[24] Perez-Ruiz F (2009). Treating to target: a strategy to cure gout. Rheumatology (Oxford).

[25] Halpern R, Fuldeore MJ, Mody RR, Patel PA, Mikuls TR (2009). The effect of serum urate on gout flares and their associated costs: an administrative claims analysis. J Clin Rheumatol.

[26] Annemans L, Spaepen E, Gaskin M (2008). Gout in the UK and Germany: prevalence, comorbidities and management in general practice 2000–2005. Ann Rheum Dis.

[27] Abhishek A, Valdes AM, Zhang W, Doherty M (2016). Association of serum uric acid and disease duration with frequent gout attacks: a case-control study. Arthritis Care Res (Hoboken).

[28] Fokter SK, Repse-Fokter A (2010). Acute gouty arthritis in a patient after total knee arthroplasty. Wien Klin Wochenschr.

[29] Dang L, Kim JK, Lee KB (2019). Crystal-induced arthritis after total ankle arthroplasty. J Am Podiatr Med Assoc.

[30] Sanders B, Abdulfatah M, Aljuaid M, Tawhari I (2019). Polyarticular septic arthritis due to non-typeable haemophilus influenzae with concomitant new-onset acute gouty arthritis. J Investig Med High Impact Case Rep.

[31] Yu KH, Luo SF, Liou LB (2003). Concomitant septic and gouty arthritis–an analysis of 30 cases. Rheumatology (Oxford).

