# Supplementary material for: Acute gout attacks during the perioperative period and risk factors of recurrence after orthopedic surgery among untreated gout patients
Source: J Orthop Surg Res. 2023 Jan 23;18:61. doi: 10.1186/s13018-023-03536-8 (PMC9869566; doi:10.1186/s13018-023-03536-8)
Supplement: Supplementary file 1 — Additional file 1. Supplement Table 1. Effects of different uric acid groups on gout attack. [file 13018_2023_3536_MOESM1_ESM.docx]

Table S1. Effects of different uric acid groups on gout attack.

| Category | gout recurrence | | P |
| --- | --- | --- | --- |
|  | Yes | No |  |
| Group 1: |  |  | 0.005 |
| ≤420μmol/L | 3 (88.5) | 23 (11.5) |  |
| > 420μmol/L | 17 (44.7) | 21 (55.3) |  |
| Group 2: |  |  | 0.013 |
| ≤360μmol/L | 2 (10.0) | 18 (90.0) |  |
| > 360μmol/L | 18 (40.9) | 26 (59.1) |  |
| Group 3: |  |  | 0.160 |
| ≤300μmol/L | 1 (11.1) | 8 (88.9) |  |
| > 300μmol/L | 19 (34.5) | 36 (65.5) |  |
